# The Effectiveness of Compartmentalized Bone Graft Sponges Made Using Complementary Bone Graft Materials and Succinylated Chitosan Hydrogels

**DOI:** 10.3390/biomedicines9121765

**Published:** 2021-11-25

**Authors:** Jae Seo Lee, Hyo-Sung Kim, Haram Nah, Sang Jin Lee, Ho-Jin Moon, Jae Beum Bang, Jung Bok Lee, Sun Hee Do, Il Keun Kwon, Dong Nyoung Heo

**Affiliations:** 1 Department of Dentistry, Graduate School, Kyung Hee University, 26 Kyungheedae-ro, Dongdaemun-gu, Seoul 02447, Korea; leejaeseo@khu.ac.kr (J.S.L.); hrnah@khu.ac.kr (H.N.); 2 Department of Clinical Pathology, College of Veterinary Medicine, Konkuk University, Seoul 05029, Korea; w6373515@naver.com (H.-S.K.); shdo@konkuk.ac.kr (S.H.D.); 3 Department of Dental Materials, School of Dentistry, Kyung Hee University, 26 Kyungheedae-ro, Dongdaemun-gu, Seoul 02447, Korea; leesangjin@khu.ac.kr (S.J.L.); 3216@khu.ac.kr (H.-J.M.); 4 Department of Dental Education, School of Dentistry, Kyung Hee University, 26 Kyungheedae-ro, Dongdaemum-gu, Seoul 02447, Korea; bangjb@khu.ac.kr; 5 Department of Biological Science, Sookmyung Women’s University, Cheongpa-ro 47-gil 100 (Cheongpa-dong 2(i)-ga), Yongsan-gu, Seoul 04310, Korea; jungboklee@sookmyung.ac.kr

**Keywords:** natural polymer-based hydrogel, chitosan, bone graft, succinylation, bone tissue engineering

## Abstract

Bone defects can occur from many causes, including disease or trauma. Bone graft materials (BGMs) have been used to fill damaged areas for the reconstruction of diseased bone tissues since they are cost effective and readily available. However, BGMs quickly disperse around the tissue area, which ultimately leads to it migrating away from the defect after transplantation. We tested chitosan hydrogels as a useful carrier to hold BGMs in the transplantation area. In this study, we synthesized succinylated chitosan (SCS)-based hydrogels with a high decomposition rate and excellent biocompatibility. We confirmed that BGMs were well distributed inside the SCS hydrogel. The SCS-B hydrogel showed a decrease in mechanical properties, such as compressive strength and Young’s modulus, as the succinylation rate increased. SCS-B hydrogels also exhibited a high cell growth rate and bone differentiation rate. Moreover, the in vivo results showed that the SCS hydrogel resorbed into the surrounding tissues while maintaining the BGMs in the transplantation area for up to 6 weeks. These data support the idea that SCS hydrogel can be useful as a bioactive drug carrier for a broad range of biomedical applications.

## 1. Introduction

Major bone defects commonly occur in clinical settings due to traumatic accidents, metabolic diseases, osteoclastic tumors, or cancer resection [1]. These bone defects often lead to serious subsequent infections, complications, and sequelae that can lead to long-term gait disturbance [2]. In the majority of cases, bone grafts are performed either using the patient’s own or donor’s bone [3]. However, donor bone extraction is difficult and is often accompanied by donor site damage that requires secondary surgery to repair. Demineralized bone matrix (DBM) is a popular bone substitute that is widely used as one of the most common allografts. DBM is a part of the freeze-dried allogeneic bone that is composed of insoluble collagen and protein. It also contains a low concentration of bone morphogenetic protein [4]. However, it is not easy to obtain raw materials. Furthermore, some clinicians and patients are still wary of antigenicity and infection due to human immunodeficiency virus (HIV) or other pathogens. There is also a problem of wide variation depending on the manufacturing method and the tissue banks [5]. As compared with DBM, synthetic bone graft materials (BGMs) are safer and have more consistent results. Additionally, BGMs provide for osteoconduction and are safe against disease and viruses. Moreover, these materials have a long shelf life and remain stable providing for bone ingrowth. The latest trend in clinical usage is to apply lower HA and higher β-TCP ratios, due to beneficial cell proliferation and differentiation in the early stages [6]. Nevertheless, excessive absorption of β-TCP during bone defect repair adversely affects bone regeneration. BGMs also commonly migrate within a short time period after being deposited [7] and act to break the original bone pore structure during implantation and regrowth. This can lead to functional impairment. To prevent BGMs from escaping the defect site, many researchers have developed biomaterial-based scaffolds to hold the BGMs in place [8]. 

Of the various kinds of scaffolds, hydrogel types are the most suitable form to protect encapsulated biological materials in large quantities and physically hold them firmly together [9]. Hydrogel-type scaffolds are difficult to sterilize due to their soft nature, 3D network structure, and water content. Conversely, sponge-type scaffolds can be stored for a long time and do not present problems for sterilization [10]. Within a few seconds of placing the scaffold in blood or water, sponge-type scaffolds will rehydrate and become hydrogels. With respect to materials used in common hydrogels, chitosan is a promising biocompatible material that has great protein adsorption [11,12,13]. Despite these advantages, chitosan by itself has poor solubility, weak mechanical properties, and lacks bioactivity. These properties reduce its usefulness for bone tissue engineering. However, the incorporation of BGMs into the chitosan hydrogel provides for a scaffold in which the components act in a manner that each other to mechanical properties, bone regeneration ability, and control of degradation rate. Succinylated modifications can be utilized to improve chitosan solubility. In our previous study, succinylated chitosan (SCS) was ion-crosslinked using glucose-6-phosphate (G6P) [14]. We hypothesized that, because SCS hydrogel-based sponges have a rapid degradation, they would be good delivery materials to specifically locate BGMs into the bone defect areas. The major aim of this study was to fabricate a complex complimentary sponge composed of SCS and BGMs (SCS-B) containing potent osteogenic activity. We believe that our approach of BGMs delivery using SCS-B will demonstrate potential efficacy for repairing bone defects by promoting bone regeneration.

## 2. Materials and Methods

### 2.1. Materials

Low-molecular-weight chitosan (MW: 50,000–190,000 Da), 4-dimethylaminopyridine (>98%), D-glucose 6-phosphate sodium salt (G6P), and succinic anhydride (>99%) were purchased from Sigma Aldrich (St Louis, MO, USA). Acetic acid was purchased from Junsei Chemical Co. (Ltd., Japan). Human adipose tissue-derived mesenchymal stem cells (hAT-MSCs), CEFOgro^TM^ ADMSC human adipose tissue-derived MSC growth medium (GM), Osteo-differentiation medium (OM), and supplement (10% FBS, 0.02% Penicillin and Streptomycin) were purchased from CEFO CO (Seoul, Korea). Synthetic bone graft (Q-Oss+) was purchased from Osstem (Seoul, Korea). All reagents and solvents were used as received without further purification.

### 2.2. Preparation of Bone Graft Materials (BGMs) Loaded with Succinylated Chitosan (SCS)-Based Hydrogel (SCS-B)

SCS was synthesized as previously reported, and varying degrees of succinylation were created by adjusting the An/Am ratio [14]. SCS was prepared at three levels of succinylation based on An/Am ratios of 0.35 (SCS-L), 0.5 (SCS-M), and 0.7 (SCS-H). These were all prepared as 2.5% *w*/*v* solutions in deionized water (DW). The G6P solution (2 mg/uL) was prepared at room temperature overnight using DW. BGMs were placed in each SCS solution and dispersed evenly. Then, the G6P solution was added to each SCS solution at a volume ratio of 2:1 (SCS solution:G6P solution) to crosslink the gel. The hydrogels were prepared as a film with a thickness of 1 mm and shaped into an 8 mm diameter disk using a biopsy punch. The hydrogels were subsequently freeze-dried.

### 2.3. Characterizations of Synthesized SCS-B

SCS-B was observed using a scanning electron microscope (SEM, S-4700, Hitachi, Japan) at an acceleration voltage of 15 kV. All samples were dried at room temperature, and then sputter-coated with gold using an IB-3 coater (Eiko, Japan) for 10 min. The images were evaluated using Eyeview-Analyzer (Digiplus, Korea). The various SCS-B sample disks were evaluated using a rotating rheometer (Anton Paar, Graz, Austria) in oscillation mode with a plate–plate geometry of 8 mm diameter and a 1 mm gap. The samples were measured for storage elastic modulus (G′) and loss modulus (G″) to determine viscoelastic behavior. A frequency sweep was carried out in the range of 0.1–10 Hz, at a constant strain of 1%. The calculation formula of G′ and G″ is as follows:

$$G^{\prime }=\frac{\sigma_{0}}{\varepsilon_{0}}\cos\delta ,\;G^{\prime \prime }=\frac{\sigma_{0}}{\varepsilon_{0}}\sin\delta$$

To measure compressive strength, SCS-B samples were prepared in the form of a cylinder with 10 mm diameter and 10 mm height. These were tested using a mechanical tester at a compression rate of 1 mm/min. The anatase and rutile phases were characterized by X-ray diffraction (XRD) (D8 Advance, Bruker, Billerica, MA, USA). The XRD analysis of SCS-L, BGMs, and SCS-LB was performed at 40 kV and 40 mA in the range of 10–70° x.

### 2.4. Cell Viability and Proliferation

In all experiments, hydrogels were placed in 48-well plates, washed with sterilized phosphate-buffered saline (PBS), and placed in a culture medium, which was changed every 2 days. To evaluate biocompatibility, hAT-MSCs were seeded at a density of 1 × 10^4^ on the hydrogels. To evaluate the cell viability, all groups were treated with a calcein-AM/ethidium homodimer-1 (EthD-1) LIVE/DEAD assay kit (Invitrogen, CA, USA) at 24 and 48 h. The stained cells were observed and imaged using an EVOS^®^ FL Auto Cell Imaging System (Thermo Fisher Scientific, Waltham, MA, USA). ImageJ (National Institutes of Health, Bethesda, MD, USA) was used to quantify the total number of live and dead cells within samples. Cell proliferation was evaluated after 1, 3, and 5 days of culture at 37 °C in a 5% CO_2_ atmosphere. For this, 100 μL of CCK (Dojindo Laboratories, Kumamoto, Japan) solution was added to each well, and the plate was incubated for 1.5 h. The absorbance was measured at 450 nm using a Benchmark Plus microplate spectrophotometer (BR170-6930, Bio-Rad, Hercules, CA, USA).

### 2.5. In Vitro Osteogenic Differentiation

After 1 day of culture by GM, the medium was changed to OM. Cell culture continued with samples drawn at 5, 10, and 15 days. The total cellular protein was extracted using cell lysis buffer, and protein concentration was determined by the Bradford method using bovine serum albumin (BSA) as a standard (Bradford, 1976). The concentration of protein (in mg/mL) was standardized using the equation y = 0.0235 x + 0.2829 with an R^2^ value of 0.9946, where y is absorbance and x is concentration. In a standard reaction, 50 µL of each BSA concentration and the sample were mixed with 1.5 mL of Bradford reagent and allowed to stand at RT for 5 min, and then absorbance was measured at 595 nm. Alkaline phosphatase (ALP) activity was determined for hAT-MSCs cultured at pre-determined time periods. At each time period, the cells were washed with PBS and lysed with 3× RIPA buffer at 4 °C for 1 h. The lysed hAT-MSCs were centrifuged at 10,000 rpm for 10 min, and the supernatant was reacted with p-nitrophenol phosphate (pNPP, Sigma Aldrich) in a 37 °C incubator for 30 min. The level of p-nitrophenol production was measured using a plate reader at 405 nm wavelength. For the alizarin red staining (ARS), the mineralized matrix nodules formed by hAT-MSCs were evaluated. On days 10 and 15, the cells were washed 3 times with PBS, fixed in 4% paraformaldehyde for 30 min, and incubated with alizarin red for 10 min at room temperature. After that, the non-bound alizarin red stain was removed by washing 3 times with distilled water. For quantification of the mineralization, the mineralized matrix nodules were dissolved in 10% (*w*/*v*) cetylpyridinium chloride (Aladdin), and absorbance was measured at 540 nm using a microplate reader. The concentration of ARS (mM) was determined using the equation y = 0.4508 x + 0.0067 with an R^2^ value of 0.9989, where y is absorbance and x is concentration.

### 2.6. In Vivo Study

Male 8-week-old Sprague Dawley rats (*n* = 36), weighing an average of 200 g each, were purchased from Orient Bio (Seongnam, Gyeonggi-do, Korea). The animals were housed under a controlled temperature of 23 °C ± 2 °C, 50% ± 5% humidity, and a 12 h light–dark cycle. All experimental procedures were performed in compliance with protocols approved by the Institutional Animal Care and Use Committee of Konkuk University. (KU19061; approved date, 8 October 2018). For implantation of hydrogel scaffold, the surgical area was clipped and aseptically prepared with povidone-iodine. General anesthesia was induced by intramuscular injection of xylazine HCL (Bayer Korea, Gyeonggi-do, Korea) and maintained with inhalation of 1.5–2% isoflurane (Hana Pharm, Gyeonggi-do, Korea) and oxygen. A critical-sized calvarial defect with a diameter of 6 mm was created using a trephine bur. The animals were randomly allocated into six groups as follows: negative control group with calvarial defect only without implantation (control); Bone graft group with implantation of bone powder (BGMs); low succinylation (the An/Am ratio of 0.35; SCS-L); the An/Am ratio of succinylation of 0.35 (SCS-LB); 0.5 (SCS-MB); 0.7 (SCS-HB) with an equivalent amount of BGMs. For implantation, 40 mg of β-TCP was grafted, and SCS-L and SCS-B samples were prepared in the form of disks with a diameter of 6 mm and a thickness of 1 mm. The animals were sacrificed at 6 weeks after implantation. The calvaria tissues were harvested and fixed in 10% neutralized formalin for further analysis.

### 2.7. Micro-Computed Tomography (μ-CT) Analysis

Six weeks after implantation, calvariae were harvested for μ-CT examination. Examinations were carried out by using a SkyScan1173 (Bruker, Kontich, Belgium) at 13.85 μm resolution with a source voltage of 130 kVp, current of 60 μA, and an exposure time of 500 ms. The degree of bone density based on μ-CT was quantified using mean gray values and standard deviation of the region of interest. The reconstruction was performed by NRecon software (Bruker, Kontich, Belgium). For quantitative analysis, new bone volume (mm^3^) and percent bone volume ratio (bone volume/tissue volume, %) were measured. For histologic analysis, the calvaria samples were decalcified with 10% EDTA solution. After a routine processing procedure, samples were embedded in paraffin and cut into 4 μm sections. The sections were stained with hematoxylin and eosin (H&E), toluidine blue, and Masson’s trichrome for microscopic examination.

### 2.8. Statistical Analyses

All tests were performed in triplicate and statistical analysis was performed using GraphPad^®^ Prism (GraphPad Software, San Diego, CA, USA). Statistical comparisons were made by one-way analysis of variance, with post hoc comparisons by Tukey’s test as appropriate or two-way analysis. Statistically significant values are defined as * *p* < 0.05, ** *p* < 0.01, *** *p* < 0.001.

## 3. Results and Discussion

### 3.1. Preparation and Characterization of SCS-B

**Figure 1:**
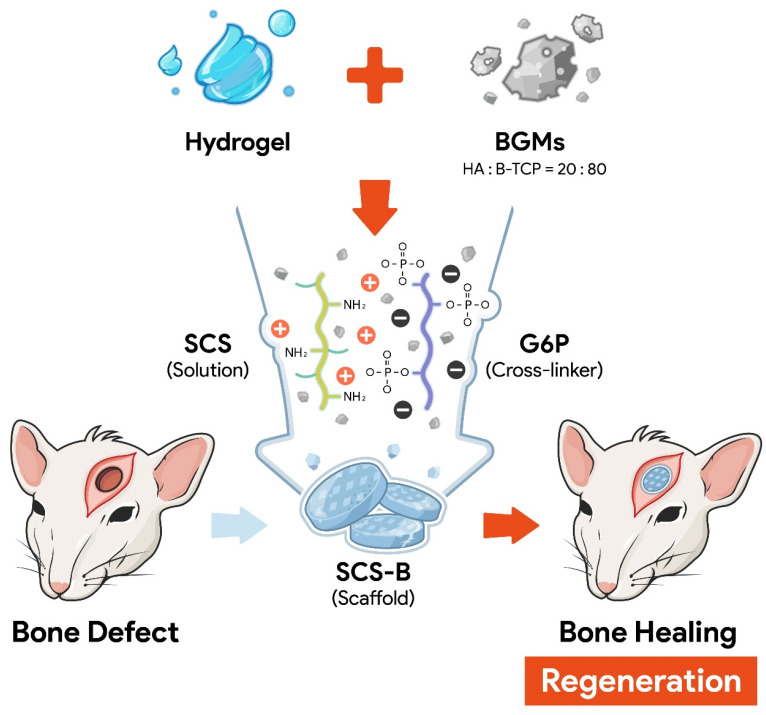
Schematic diagram of compartmentalized SCS-B hydrogel with enhanced integration and bone healing properties.

**Figure 2:**
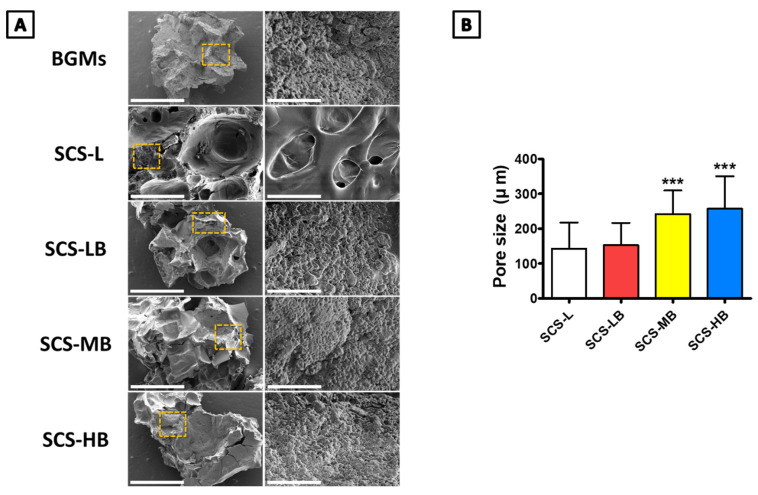
(**A**) The SEM images of cross sections of freeze-dried BGMs, SCS-L, SCS-LB, SCS-MB, and SCS-HB hydrogel sponges (scale bars = 40 μm (left), 10 μm (right)); (**B**) porosity size of the SCS-L, SCS-LB, SCS-MB, and SCS-HB hydrogel sponges. “***” indicates significant difference of *p* < 0.001.

**Figure 3:**
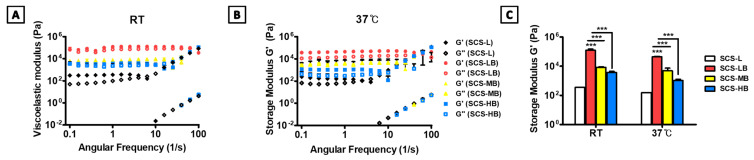
The rheological behavior depending on temperature of the SCS-L, SCS-LB, SCS-MB, and SCS-HB hydrogel sponges with different amounts of succinylation: (**A**) the rheological properties at RT; (**B**) the rheological properties at 37 °C (body temperature); (**C**) comparison of rheological behavior with temperature. “***” indicates significant difference of *p* < 0.001.

**Figure 4:**
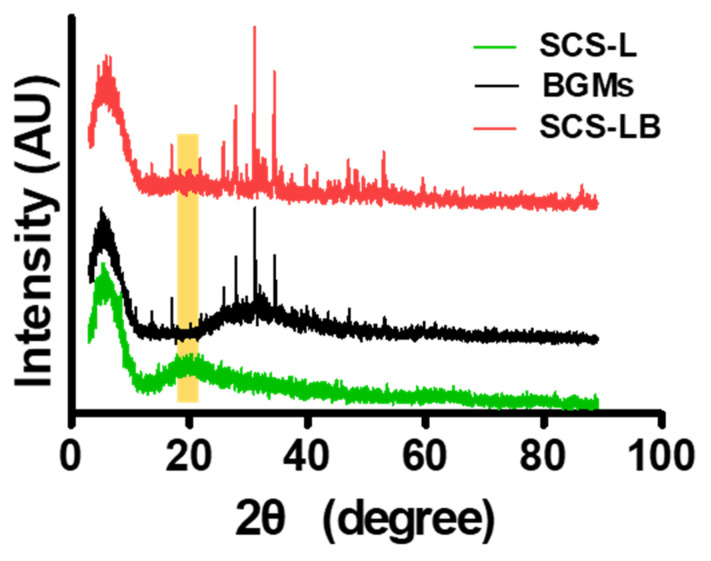
XRD patterns of the BGMs, SCS-L, and SCS-LB hydrogel sponges.

**Figure 5:**
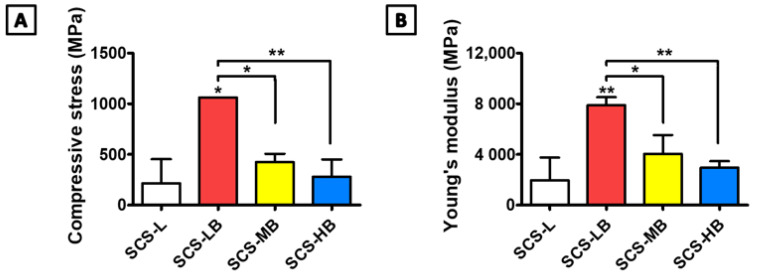
The mechanical properties of the SCS-L, SCS-LB, SCS-MB, and SCS-HB hydrogel sponges and the corresponding (**A**) compression strength and (**B**) elastic modulus. “*” indicates significant difference of *p* < 0.05. “**” indicates significant difference of *p* < 0.01.

Figure 1 shows a schematic representation of the entire process for forming BGMs-loaded SCS (SCS-B). SCS was fabricated by mixing glucose-6-phosphate (G6P) as a cross-linker [15] with SCS hydrogels containing different chitosan succinylation rates: low-succinylated chitosan (SCS-L), medium-succinylated chitosan (SCS-M), and high-succinylated chitosan (SCS-H). At the same time, an equivalent amount of BGMs was added to the mixed solution with SCS and G6P to make a homogeneous composite. In this study, these are designated as SCS-LB (BGMs-loaded SCS-L), SCS-MB (BGMs-loaded SCS-M), and SCS-HB (BGMs-loaded SCS-H) for different succinylation rates of SCS. The developed SCS-based hydrogels provide mechanisms for controlled biomolecules delivery applications with the potential for temperature and pH sensitivity [14]. In this study, BGMs were encapsulated in SCS hydrogels with various levels of succinylation. Additionally, this scaffold was made in the form of a compartmentalized sponge for easy implantation. The important point in the SCS-B process is that SCS plays a role in preventing damage to the intrinsic pores of BGMs in the initial state, as well as bringing together the hydrogel with the BGMs. Here, the lyophilized sponge maintains its own intrinsic shape and swells to restore its original, hydrated state within a few seconds as soon as it is transplanted. SEM analysis showed porous, sponge-like structures in the SCS-B after freeze drying (Figure 2A). The porosity of the SCS-B hydrogels was reduced, suggesting BGMs were homogeneously mixed in with the SCS. The SCS-L had intrinsic pores, while SCS-LB, SCS-MB, and SCS-HB pores were filled with equal proportions of BGMs and were determined to have pore size differences. In the case of SCS-LB, it was confirmed that the pores were smaller than that of SCS-MB and SCS-HB (Figure 2B). This is because of the many remaining amine groups that act to increase ionic bonding. Furthermore, this hydrogel sponge has been shown to keep its shape intact. This has a decisive effect on final mechanical and material properties. The effects of temperature on mechanical properties are shown in Figure 3. We measured viscoelasticity at room temperature (RT) and 37 °C (human body temperature). Both G′ and G″ at 37 °C were decreased, as compared with RT, suggesting a lower mechanical stiffness. This is most likely because an increase in temperature causes an increase in the molecular motion of the solution. Subsequently, the intramolecular networks increased the mobility of charged ions and accelerated the electrostatic interactions [16]. From lowest to highest, the mechanical stiffness increased in the order of SCS-HB, SCS-MB, SCS-LB, and SCS-L. Among these, SCS-LB exhibited the highest storage modulus, suggesting the highest mechanical stiffness. This trend suggests cross-linking density and stiffness are associated. Indeed, SCS-LB, with a higher amount of amine groups, showed significantly higher cross-linking efficiency than the other groups. The rheological measurements are related to the porous structure and mechanical properties of the hydrogel as well [17]. In addition, these data are fully consistent with previous SEM micrographs of microstructure morphology (Figure 2). The chemical composition of each specimen was analyzed using X-ray diffraction (XRD). As shown in Figure 4, the major proportion of BGM peaks was observed at 17°, 25°, 27°, 31°, and the 34°. The SCS-L peak was observed at 20°. Furthermore, all peaks related to BGMs and SCS-L appeared in the SCS-LB sample together. These results suggest that SCS-LB was evenly blended with SCS-L and BGMs. Moreover, these data confirm that the gel and BGMs were well dispersed from the previous microstructure morphology (Figure 2). Next, we compared the mechanical properties of SCS-L, SCS-LB, SCS-MB, and SCS-HB (Figure 5). As the succinylation rate increased, the hydrogel compressive stress and Young’s modulus decreased. This is likely due to the decreased SCS hydrogel cross-linking density. The increased chitosan succinylation rate increases SCS solubility with decreased polymer chain length, which diminishes the physical entanglement between chitosan molecules [16]. Overall, these results demonstrate the success of SCS-B to load BGMs.

### 3.2. In Vitro SCS-B Cell Viability and Osteogenic Differentiation

**Figure 6:**
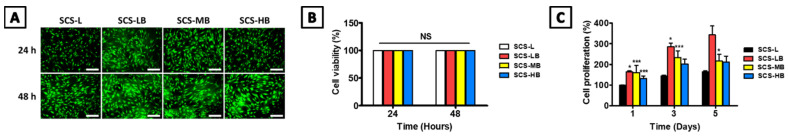
Evaluation of cell viability, proliferation of the SCS-L, SCS-LB, SCS-MB, and SCS-HB hydrogel sponges: (**A**) live/dead staining images for evaluating cell adhesion (scale bars = 500 μm); (**B**) cell viability rate obtained from the quantitative analysis of live/dead staining images (*n* = 6); (**C**) cell proliferation rate by CCK-8 (*n* = 3). “*” indicates significant difference of *p* < 0.05. “***” indicates significant difference of *p* < 0.001.

To evaluate hydrogel cytotoxicity, hAT-MSCs were seeded on the surface of SCS-L, SCS-LB, SCS-MB, and SCS-HB for direct cell-to-materials contact. This is similar to SCS-B located in a defective area of the bone. First, live/dead staining was performed to assess the biocompatibility (Figure 6A). Most of the hAT-MSCs were bright green, indicating no distinct dead cells. hAT-MSCs in all groups exhibited more than 99% viability after 24 h and 48 h. SCS-LB cell viability (99.95%) was slightly higher than SCS-L (99.94%), SCS-MB (99.94%) and SCS-HB (99.91%) after 48 h (Figure 6B). Therefore, all the experimental groups had no apparent cytotoxicity or problems with cell adhesion and proliferation during the initial period. In addition, the proliferation rate was evaluated in relationship to the different succinylation rates. No cytotoxicity was observed in all groups during the cell proliferation study (Figure 6C). All three hydrogels increased cell proliferation over the course of time. However, SCS-HB, SCS-MB, and SCS-L had a lower proliferation rate than SCS-LB at all time points. This effect in cell viability is likely due to the number of carboxyl groups in the hydrogel. In the case of increased succinylation and pH oscillations, the remaining few cations of chitosan were difficult to bind to the anionic macromolecules that regulate the activity of cytokines and growth factors, such as glycosaminoglycans (GAGs) [18]. These points can also affect osteogenic differentiation. Accordingly, the appropriate rate of succinylation should be selected to ensure good cellular compatibility [19]. 

**Figure 7:**
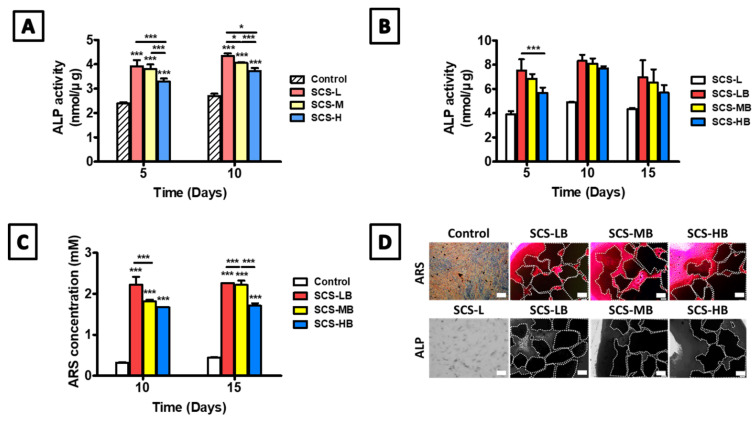
Evaluation of bone differentiation of the SCS-L, SCS-LB, SCS-MB, and SCS-HB hydrogel sponges: (**A**) expression of alkaline phosphatase activity (ALP) at hydrogel in accordance with different succinylation rates (*n* = 4); (**B**) expression of alkaline phosphatase activity at hydrogel sponge loaded with BGMs (*n* = 4); (**C**) quantification of mineralization by alizarin red staining (ARS) (*n* = 4); (**D**) ALP at 10 days and ARS staining at 15 days (Scale bar = 500 μm). “*” indicates significant difference of *p* < 0.05. “***” indicates significant difference of *p* < 0.001.

Chitosan is a preferred material for bone tissue repair due to its physicochemical and biological properties [20]. Chitosan enhances cell adhesion, proliferation, as well as osteogenic differentiation and mineralization [21,22]. The porous structure of chitosan has an effect on osteoconduction [23], and its intrinsic cationic property allows the connection of growth factors [21]. Therefore, we further evaluated the ability of osteogenic differentiation of hAT-MSCs with exclusively SCS hydrogel using alkaline phosphatase (ALP) activity as an early indicator of osteogenic differentiation (Figure 7A). As compared with control, a significant increase in ALP production was observed in SCS-L after 5 and 10 days of culture. ALP activity has been known to decrease after reaching its peak at about 10–14 days. After this, calcium minerals start to accumulate in the ECM [24]. The hAT-MSCs of ALP activity level was significantly increased in the order of SCS-H, SCS-M, and SCS-L. This gradient effect may be attributed to the number of remaining carboxyl groups after degradation due to acidification, which increases cell cytotoxicity and lowers cell differentiation [25]. When the gels were loaded with BGMs, all of SCS-B had higher ALP expressions, as compared with the SCS-L hydrogel after 5 days (Figure 7B). Interestingly, all groups showed higher ALP expression after 10 days of culture, as compared with the control. At this time, the hydrogel had already attained bone matrix maturation and mineralization after induction of osteogenic differentiation. In response to rapid ALP expression, both mineralization and ECM protein production are induced during bone development. Subsequently, calcium deposition in the ECM is important to the final stage of bone formation or differentiation into bone cells. Two weeks after the induction of bone formation, calcium content was quantified after 10 and 15 days by alizarin red staining (Figure 7C). When compared with the control group, all SCS-B groups formed higher amounts of calcium deposits for up to 15 days. The increase in calcium was elevated more significantly in the SCS-LB group than in other groups. As shown in Figure 7D, the ALP and ARS staining results are similar to our prior evaluation. There is a difference in the concentration of succinylation, but the amount of BGMs is the same. In the case of SCS-HB, it is expected that decomposition has begun. However, this is attributed to only a difference as compared with control cells on the plate, not on the SCS-L in ARS staining. The succinylation rate also influenced the biological response of the cells and had a greater effect on osteogenic differentiation. These data suggest that SCS-B is an attractive material to construct bone scaffolds from as it has excellent biocompatibility and acts to preserve differentiated bone phenotypes. 

### 3.3. In Vivo Bone Regeneration of SCS-B

**Figure 8:**
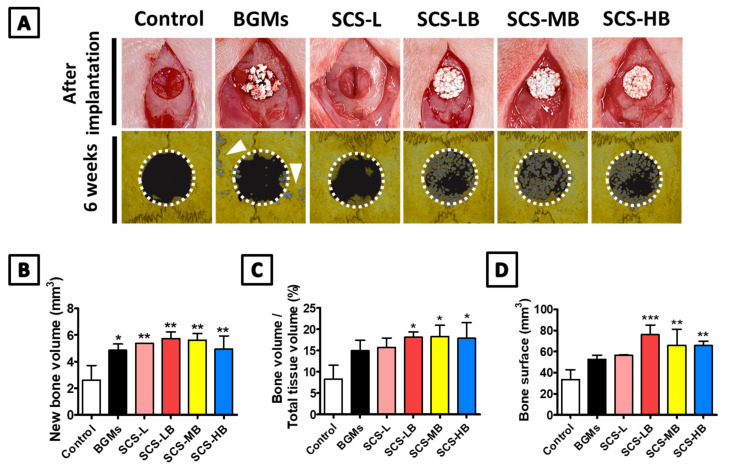
In vivo characterization of new bone tissue formation after implantation of control (defect only), BGMs, SCS-L, SCS-LB, SCS-MB, and SCS-HB hydrogel sponges: (**A**) representative μCT image of each group after 6 weeks of implantation. Quantification of (**B**) newly formed bone volume (mm^3^), (**C**) bone volume per tissue volume (BV/TV, %), and (**D**) bone surface area (mm^3^) “*” indicates significant difference of *p* < 0.05. “**” indicates significant difference of *p* < 0.01. “***” indicates significant difference of *p* < 0.001.

**Figure 9:**
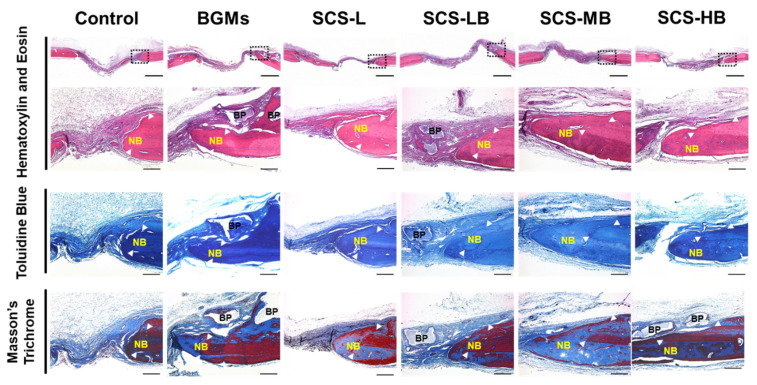
Histology of rat skin sections stained with control (defect only), BGMs, SCS-L, SCS-LB, SCS-MB, and SCS-HB hydrogel sponges: hematoxylin and eosin [H&E, scale bars = 1 mm (upper row), 200 μm (lower row)], toluidine blue, and Masson's trichrome after sacrifice of various hydrogels for 8 weeks (NB: new bone; BP: bone powders, scale bars = 200 μm).

Lyophilized SCS-B was implanted in a rat calvaria defect model for treating bone defects in vivo. Figure 8A displays the sponge-type scaffold after implantation. The scaffold mixed well with blood with good wettability. With its porous structure, lyophilized SCS-L and SCS-B could be quickly reswelled to their original shape and filled the adequate area rapidly. Defect reconstruction images were taken using micro-computed tomography (μCT) imaging at 6 weeks after implantation (Figure 8A). With the results of multiple micro-CTs in Figure S1, it is easy to understand the findings at a glance. All SCS-B groups showed increased bone formation, as compared with the control. However, bone formation was not complete. In SCS-L and SCS-B groups, new bones started to form around the edges of the defect and gradually regenerated toward the center. Bone healing is usually accomplished from the edge of the defect and at the periosteum. When a defect occurs, both the dura mater, which helps bone formation, and the periosteum, which aids blood supply, are severely damaged. Therefore, it is important to start the healing from the edges [26]. Accordingly, in the early stages, a selection of materials and shapes is crucial to provide for a large contact area with the edge of the defect [27]. However, there is almost no bone formation in the target area in the control and BGMs groups. In the BGMs groups, the majority of BGMs had migrated away from the defect and could not be readily located. Most SCS-B groups, which use the hydrogel to prevent BGMs migration, presented regenerated bone by 6 weeks. Both SCS-LB and SCS-MB were similar to each other in their ability to maintain BGMs and regenerate new bone. SCS-HB had BGMs migration due to its low crosslinking and relatively rapid degradation. Bone formation was further confirmed by examining the amount of new bone volume (NBV), the ratio of bone surface density to total tissue volume (BV/TV), and the amount of new bone surface. As shown in Figure 8B, SCS-LB exhibited the highest NBV in the groups, about a twofold increase in comparison with the control groups. BV/TV in the all SCS-B groups was higher than in the control groups (8.25% ± 3.30% and 14.95% ± 2.51% in the control and BGMs, respectively) (Figure 8C). For the new bone surface, the SCS-LB group showed significantly higher healing of the defect area among the SCS-B groups (Figure 8D). This is attributed to the osteogenic differentiation binding capacity with SCS hydrogel for new bone surfaces. Therefore, we concluded that SCS-L is an effective scaffold to help bone regeneration. Histologic analysis was conducted to confirm μCT data on calvarial bone regeneration. The original bone and newly formed bone were differentiated and highlighted using three staining methods, hematoxylin and eosin (H&E), toluidine blue, and Masson's trichrome (Figure 9). In the control group, only a small amount of new bone, resembling periosteal reaction, was found on the edge of the defect. The amount of new bone seemed elevated by bone powder in the BGMs group; however, the BGMs were not located between the defect edges and had moved around. This causes osteogenesis in unwanted locations and also leads to reduced original bone integrity. In the SCS-L group, however, newly formed bone was sprouting out from the edge. This is attributed to the osteoconduction ability of the hydrogels. Unlike the BGMs group, the bone powder of SCS-B groups remained in the intended location in the middle of the defect, and the original calvarial bone showed an intact lamellated appearance. The immature bone in the SCS-B groups was formed around the defect edges and scattered across the bone graft. This was confirmed by histological analysis that bone regeneration of SCS-B groups was well controlled in the defect area alone and not conflicting with other, normal tissue. 

Bone allograft is the golden standard biomaterial for bone regeneration [28]. However, autologous bone graft has limitations because of its restricted supply and donor site complications. Patients who need a bone graft may not have healthy tissue due to underlying conditions such as diabetes. Furthermore, the harvesting process causes the patient to suffer from collateral damage, which limits the procedure’s utility. For these reasons, a demineralized bone matrix can be a suitable alternative. However, histologic images show that peri-implant osteolysis can occur due to the unintended distribution of bone particles [29]. The results from this in vivo study showed that SCS-B can be a solution for successful implantation of bone powder, holding the bone particles in the area of the defect and restricting unwanted side effects of bone powder. In the absence of BGMs, SCS-L does not readily maintain the initial volume until the bone regenerates. However, BGMs in SCS-B are not quickly decomposed and maintain their structure for up to 6 weeks. This improves SCS-B’s ability to aid bone regeneration in the later stages of healing. Consequently, chitosan combined with BGMs provides for stable mechanical properties and sufficient bone-bonding ability. Additionally, chitosan has an innate antibacterial activity that prevents infections that can lead to implant failure. In addition, BGMs both protect the chitosan polymer from enzymatic degradation and neutralize acidification caused by chitosan degradation. This allows the scaffold to last longer and provides for better ingrowth of the bone and eventual formation of hard tissue [30,31]. There is an increasing clinical need for injectable biomaterials. These can be adapted to various platforms and can enable minimally invasive surgery [32]. SCS-B has flexibility for use as a carrier for precisely locating bone powder where it is desired. It also provides for a sponge that quickly hydrates to its original state when it encounters bodily fluid. As it has osteoinductive and osteoconductive properties, it is a suitable biomaterial for bone regeneration.

## 4. Conclusions

We have successfully fabricated a biocompatible chitosan-based hydrogel composed of SCS and BGMs. SCS-B exhibited a porous structure, enhanced mechanical properties, and controllable release rate in accordance with the succinylation rate. Furthermore, SCS-B had remarkable osteogenic differentiation in vitro. After implanting into a calvarial bone defect, SCS-B was able to maintain the proper shape and provide for bone regeneration. The results from this study indicate that SCS-based hydrogels will expand the biomedical tool kit and contribute to tissue engineering development in the future.

## Data Availability

Not applicable.

## Supplementary Materials

The following are available online at https://www.mdpi.com/article/10.3390/biomedicines9121765/s1, Figure S1: In vivo characterization of new bone tissue formation after implantation of control (defect only), BGMs, SCS-L, SCS-LB, SCS-MB, and SCS-HB hydrogel sponges: representative μCT images of each group after 6 weeks of implantation.

## Abbreviations

BGMs	Synthetic bone materials
SCS-L	Low-succinlyated chitosan
SCS-LB	BGMs-loaded SCS-L
SCS-MB	BGMs-loaded SCS-M
SCS-HB	BGMs-loaded SCS-H
