# Supplementary material for: The Effectiveness of Compartmentalized Bone Graft Sponges Made Using Complementary Bone Graft Materials and Succinylated Chitosan Hydrogels
Source: Biomedicines. 2021 Nov 25;9(12):1765. doi: 10.3390/biomedicines9121765 (PMC8698467; doi:10.3390/biomedicines9121765)
Supplement: Supplementary file 1 [file biomedicines-09-01765-s001.zip › biomedicines-1441217-supplementary.pdf]

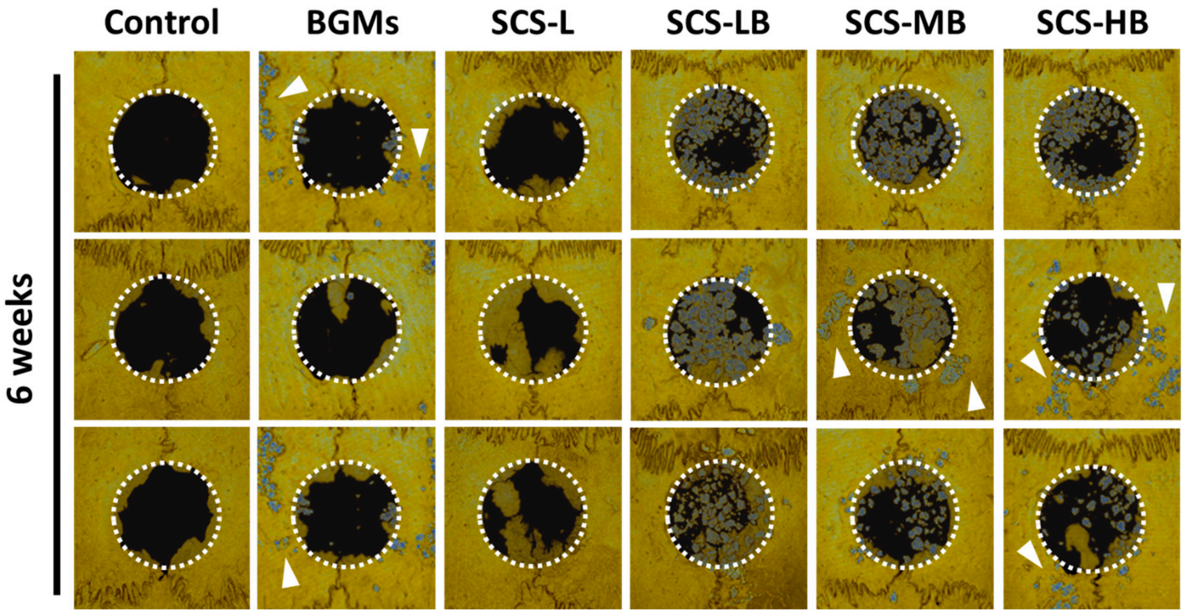

Figure S1. In vivo characterization of new bone tissue formation after implantation of control (defect only), BGMs, SCS-L, SCS-LB, SCS-MB, and SCS-HB hydrogel sponges: all μCT images of each group after 6 weeks of implantation.
